# A pentaploid-based linkage map of the ancestral octoploid strawberry *Fragaria virginiana* reveals instances of sporadic hyper-recombination

**DOI:** 10.1038/s41438-020-0308-2

**Published:** 2020-05-07

**Authors:** Thomas M. Davis, Yilong Yang, Lise L. Mahoney, Daniel C. Frailey

**Affiliations:** Department of Agriculture, Nutrition, and Food Systems, University of New Hampshire, Durham, NH 03824 USA

**Keywords:** Plant breeding, Plant genetics

## Abstract

The first high-resolution genetic linkage map of the ancestral octoploid (2n = 8x = 56) strawberry species, *Fragaria virginiana*, was constructed using segregation data obtained from a pentaploid progeny population. This novel mapping population of size 178 was generated by crossing highly heterozygous *F. virginiana* hybrid “LB48” as a paternal parent with diploid (2n = 2x = 14) *Fragaria vesca* “Hawaii 4”. The LB48 linkage map comprises 6055 markers genotyped on the Axiom® IStraw90 strawberry SNP array. The map consists of 28 linkage groups (LGs) organized into seven homoeology groups of four LGs each, and excludes a small 29th LG of undefined homoeology. One member of each homoeology group was assignable to an “A” subgenome associated with ancestral diploid *Fragaria vesca*, while no other subgenomes were defined. Despite an intriguing discrepancy within homoeology group VI, synteny comparisons with the previously published *Fragaria* ×*ananassa* DA × MO linkage map revealed substantial agreement. Following initial map construction, examination of crossover distributions revealed that six of the total 5162 (=29 chromosomes/individual × 178 individuals) chromosomes making up the data set exhibited abnormally high crossover counts, ranging from 15 to 48 crossovers per chromosome, as compared with the overall mean of 0.66 crossovers per chromosome. Each of these six hyper-recombinant (HypR) chromosomes occurred in a different LG and in a different individual. When calculated upon exclusion of the six HypR chromosomes, the canonical (i.e., broadly representative) LB48 map had 1851 loci distributed over a total map length of 1873 cM, while their inclusion increased the number of loci by 130, and the overall map length by 91 cM. Discovery of these hyper-recombinant chromosomes points to the existence of a sporadically acting mechanism that, if identified and manipulable, could be usefully harnessed for multiple purposes by geneticists and breeders.

## Introduction

The assembly of reference genomes for polyploids, such as the octoploid (2n = 8x = 56) cultivated strawberry *Fragaria* ×*ananassa*, and its immediate 8x ancestors *F. chiloensis* and *F. virginiana*, is an ongoing challenge^1,2^. Genome assemblies in many diploid and polyploid plant species have been enhanced by anchoring to genetic linkage maps^3^, and/or by the choice of haploid or double-haploid individuals as the object of the sequencing effort^4–6^. Neither anchoring nor haploid approaches to genome assembly have yet been employed in an octoploid strawberry species.

The octoploid, cultivated strawberry is subject to inbreeding depression^7^, and haploid or doubled-haploid clones have not been a readily available option in *Fragaria*. We have taken advantage of the fact that hybrid pentaploid progeny populations can be generated from certain diploid × octoploid crosses in *Fragaria*^8^. As allohaploids, these pentaploids offer potential advantages for genomic research, and can serve as “surrogate haploids” in relation to both linkage mapping and genome assembly. As a prelude to genome assembly and a basis for identifying gene–trait associations in *F. virginiana*, we have used a pentaploid progeny population to construct a high-resolution *F. virginiana* genetic linkage map. We know of no prior study in which a pentaploid progeny population has been used for linkage mapping in *Fragaria*.

As the diploid crossing partner used to generate the pentaploid progeny population, we used *F. vesca* “Hawaii 4”, an inbred hermaphrodite that universally serves as a model plant for *Fragaria* genetics/genomics, and for which a high-quality reference genome was available^9,10^. The resulting map defines the linkage groups to which a genome assembly could be anchored, and illuminates patterns of segregation distortion and genetic interference. Unexpectedly, our study has also revealed the presence of an intriguing genetic phenomenon, sporadic hyper-recombination (HypR), which if understood could be usefully harnessed in the service of plant breeding and genomic research.

As enumerated below, several octoploid strawberry linkage maps have been reported, generally consisting of the expected 28 linkage groups and displaying segregation patterns consistent with meiotic diploidization and disomic inheritance. However, the possibility of genomically localized polysomic inheritance patterns has also been suggested^11^. Most published octoploid strawberry linkage maps have been based on segregation analysis of the progenies of pseudo-testcrosses, relying on the heterozygosity of each crossing parent, and entailing the construction of separate male and female parental maps, which then may be integrated into a consensus map.

A variety of marker types have been utilized in the construction of *F*. ×*ananassa* linkage maps, with initial emphasis on AFLPs and/or SSRs^11–17^, and more recently on high-throughput methods, including targeted sequence capture^18^ and SNPs^19–21^ genotyped on the Axiom IStraw90 strawberry SNP array^21^, or by other means^22,23^. Low-resolution SSR-based linkage maps have been constructed in *F. virginiana*^24,25^ and *F. chiloensis*^25^ for the purpose of mapping sex-determination loci.

The first strawberry single-nucleotide polymorphism (SNP) genotyping array, the Axiom® IStraw90® array (Affymetrix, Inc) was designed and advanced to commercial manufacture as an initiative of the USDA RosBREED Specialty Crops (SCRI) project^21^. For the purpose of array design, SNP discovery was conducted in an octoploid germplasm panel consisting predominantly of cultivated strawberry (*F*. ×*ananassa*) varieties, accounting for 85,663 of the loci targeted by the array. An additional 3751 loci were included based on identification in ancestral diploid *F. iinumae* as a basis for linkage map construction in that species^26^. Finally, 5648 single-nucleotide sites were chosen for interrogation using a non-discovery-based (“codon-based”) approach^21^. Importantly, although the array design relied on the *F. vesca* Hawaii 4 Ver 1.1 reference genome assembly^10^, SNP discovery was not conducted in Hawaii 4 or any other *F. vesca* germplasm^21^. Our pentaploid project afforded opportunity to evaluate the performance of the array for genotyping in an octoploid other than *F*. ×*ananassa*, and for which the array was not specifically designed.

## Materials and methods

### Plant materials

*Fragaria virginiana* octoploid hybrid “LB48” (USDA germplasm accession PI 664374) was derived from a cross between two octoploid *F. virginiana* accessions: female clone “L1” (PI 660769) as the maternal parent and hermaphroditic clone “BC6” (PI 660767) as the paternal parent. L1 (subspecies *virginiana*) and BC6 (subspecies *glauca*) had been previously collected from the wild in New Hampshire and British Columbia, respectively (T.M. Davis, unpublished). The pentaploid mapping population comprised 178 F1 hybrid seedlings generated from the cross between inbred, hermaphroditic diploid *F. vesca* “Hawaii 4”, as the maternal parent, and highly heterozygous hermaphrodite LB48 as the paternal parent. On the basis of this parental pairing, the vast majority of marker segregation in the pentaploid progeny population was expected to be the product of heterozygosity in the octoploid parent, LB48, whereas Hawaii 4 served as a tester. Studied plants were clonally propagated and maintained at the Macfarlane Greenhouse facility of the New Hampshire Agricultural Experiment Station (NHAES) at the University of New Hampshire (UNH) in Durham, NH, USA.

### Ploidy confirmation by flow cytometry

Flow cytometric analyses of propidium iodide-stained nuclei from freshly harvested young leaves were performed on a BD Accuri C6 instrument as described^27^ in order to evaluate the ploidies of the putative pentaploid hybrids. For this purpose, the 1C genome sizes of the parental (LB48) and grandparental (L1 and BC6) octoploids were estimated in comparison to diploid Hawaii 4.

### SNP genotyping

For the purpose of SNP genotyping, DNA samples were prepared by the method previously described by Mahoney et al.^26^. Briefly, young, partially expanded leaves were collected and refrigerated for no more than 5 days prior to DNA extraction. DNA was isolated using the E-Z 96 Plant DNA kit (Omega Bio-tek) followed by Proteinase K treatment as required by Affymetrix, Inc. DNA samples from 178 pentaploid progeny plants and two replicate sets of the grandparental (L1 and BC6), and parental (Hawaii 4 and LB48) individuals were sent to Affymetrix for genotyping on the Axiom® IStraw90® *Fragaria* whole-genome genotyping array (henceforth referred to as the IStraw90 array), and the Affymetrix GeneTitan® genotyping system was employed following the standard operation procedures (https://downloads.thermofisher.com/Axiom_Analysis_Suite_v_4.0.1_User_Guide.pdf). The resulting signal intensity files (“CEL files”, as identified by their “.cel” file extensions) were analyzed, and genotype calling was performed using the Affymetrix Axiom Analysis Suite software application. In the output data, markers were classified into the six standard Affymetrix conversion types (performance categories): PolyHighResolution (PHR), NoMinorHom (NMH), MonoHighResolution (MHR), CallRateBelowThreshold (CRBT), OffTargetVariant (OTV), and Other^21^.

Given the nature of the employed diploid (Hawaii 4) × octoploid (LB48) cross, the progeny of which were expected to segregate on the basis of heterozygosity in LB48, employed markers were required to display two-category segregation patterns corresponding to those expected of a testcross progeny in a octoploid setting. Given the existence of alternate alleles A and B, where B is the “minor” (i.e., low dose) allele of the marker in question, such a pattern would be expected if the diploid parent marker genotype was AA, and the octoploid parent marker genotype was determined by the number of homolog pairs carrying the marker locus: i.e., either (B/A)(A/A)(A/A)(A/A), or (B/A)(A/A)(A/A), or (B/A)(A/A), or (B/A), depending upon the effective ploidy of the marker site in question^21^. In this scheme, the “minor” allele “B”, would be detected as present versus absent in the progeny, resulting in two-category segregation with clearly separated clusters and robust genotype calling. If BB, the diploid parent would contribute the marker allele to all progeny, converting the assay from presence/absence to one of rare allele dosage, which creates problematic cluster compression in a polyploidy setting^21^. A three-category segregation pattern would result if both the diploid and octoploid parent were heterozygous for the same marker.

### Segregation data analysis and linkage map development

Linkage analysis was performed using JoinMap 4.1 (Kyazma, NL), where the data set type was set as haploid (HAP). Input of genotyping data into JoinMap required conversion of Affymetrix genotyping codes to appropriate JoinMap codes. In the Affymetrix output data, four codes are used: −1 = missing data; 0 = homozygous or haploid for one allele; 2 = homozygous or haploid for the alternate allele; and 1 = heterozygous. In JoinMap, the relevant genotyping codes are *n* = missing data; *a* = haploid for one allele; and *b* = haploid for the alternate allele; while heterozygosity is not a permitted state in the haploid setting. The rubric for conversion of genotype calls from the Affymetrix format to that required for input into JoinMap is provided in Supplementary Table S1, and was designed such that marker alleles derived from grandparents L1 and BC6 would be coded as “a” and “b”, respectively, in the pentaploid progeny population.

Marker segregation resulting from heterozygosity in LB48 was nominally expected in a 1:1 presence:absence ratio, but the possibility of segregation distortion in at least some genomic regions was also anticipated, and so markers deviating from a 1:1 ratio were not excluded. Linkage clusters were calculated with a minimum logarithm of odds (LOD) score of 8.0. A series of increasingly refined linkage maps was produced using the maximum likelihood (ML) algorithm with default parameters. In order to temporarily reduce marker redundancy, if two or more markers shared the same set of progeny genotypes, initially all but one representative marker was automatically removed by JoinMap, then were later added back to the resultant linkage map using the “Assign Identical Loci to Their Groups” function.

After initial map construction, the majority of markers were in JoinMap linkage phase 0, so genotype calls of markers in the alternate phase 1 were converted by interchanging “a” and “b” genotype calls. After phase correction and map recalculation, the singleton markers, i.e., those putatively miscalled single markers that generated artifactual double crossovers^21^, were identified and converted to missing data using in-house scripts. For this purpose, the singletons were identified as those interior (nonterminal) marker calls differing from both of the nearest flanking marker calls in the same individual. After singleton correction, map construction was performed again.

In the resulting final map version, segregation distortion was detected via application of the chi-square goodness-of-fit test within JoinMap based on the 1:1 segregation ratio expected for each marker. In addition, crossover positions were identified and counted using an in-house script that detected genotypic shift (“a” to “b” or vice versa) between two adjacent marker calls, while ignoring missing data. The number of crossover points in each chromosome was then determined, and the distribution of crossovers was evaluated based upon expectations derived from the Poisson distribution^28^.

### Homoeology group and subgenomic assignments of linkage groups

Based upon its marker content, each linkage group was assigned to one of seven possible homoeology groups based upon the known positions of each IStraw90 array marker in the Hawaii 4 Ver 1.1 reference genome^10^, upon which the array was designed^21^. Within each homoeology group, analysis of the subgenomic affinities of each chromosome to the ancestral *F. vesca* and *F. iinumae* genomes was performed using the approach previously employed^19^, whereby the array markers of the SNP–SNP design category^21^ were categorized as displaying patterns of affinity of four possible types: affinity to both *F. vesca* and *F. iinumae* (YY); to *F. vesca* but not *F. iinumae* (YN); to *F. iinumae* but not *F. vesca* (NY); or to neither *F. vesca* nor *F. iinumae* (NN).

### Comparison with DA × MO cultivated strawberry (*F*. ×*ananassa*) linkage map

The map positions of markers shared between the canonical LB48 map and the previously published DA × MO *F*. ×*ananassa* linkage map^19^ were compared as a means of identifying correspondences between *F. virginiana* linkage groups and those in the DA × MO map. The Circos^29^ diagrams were generated using the Circos program version 0.69–9.

## Results

### Flow cytometry and verification of pentaploid status

In their overall morphology, all progeny of the Hawaii 4 × LB48 cross resembled the paternal parent (LB48) much more so than the morphologically distinct maternal parent (Hawaii 4), thereby providing an initial confirmation of progeny hybridity. All progeny plants displayed sexual sterility, as would be expected for odd-ploid hybrids. The flow cytometer output FL2-A readings (in parentheses) of nuclear fluorescence were obtained for diploid Hawaii 4 (47,195), LB48 (148,256), L1 (144,659), BC6 (149,265), and 100 progeny seedlings from the Hawaii 4 x LB48 cross, including progeny plant 5xAJ (95,660), which had been previously confirmed as pentaploid via mitotic chromosome count^8^. The octoploid/diploid ratios of FL2-A values ranged from 3.07 (for L1) to 3.16 (for BC6), while the 5xAJ/diploid ratio was 2.03. The FL2-A value of 95,660 for plant 5xAJ was approximately half of the sum of the Hawaii 4 and LB48 FL2-A values [(47,195 + 148,256)/2 = 97,725], as would be predicted for a Hawaii 4 x LB48 pentaploid hybrid. The 99 additional progeny seedlings randomly selected for flow cytometric test using Hawaii 4 and LB48 as comparators all yielded 1C-values consistent with pentaploidy (data not shown).

### Array genotyping in the pentaploid mapping population

**Fig. 1:**
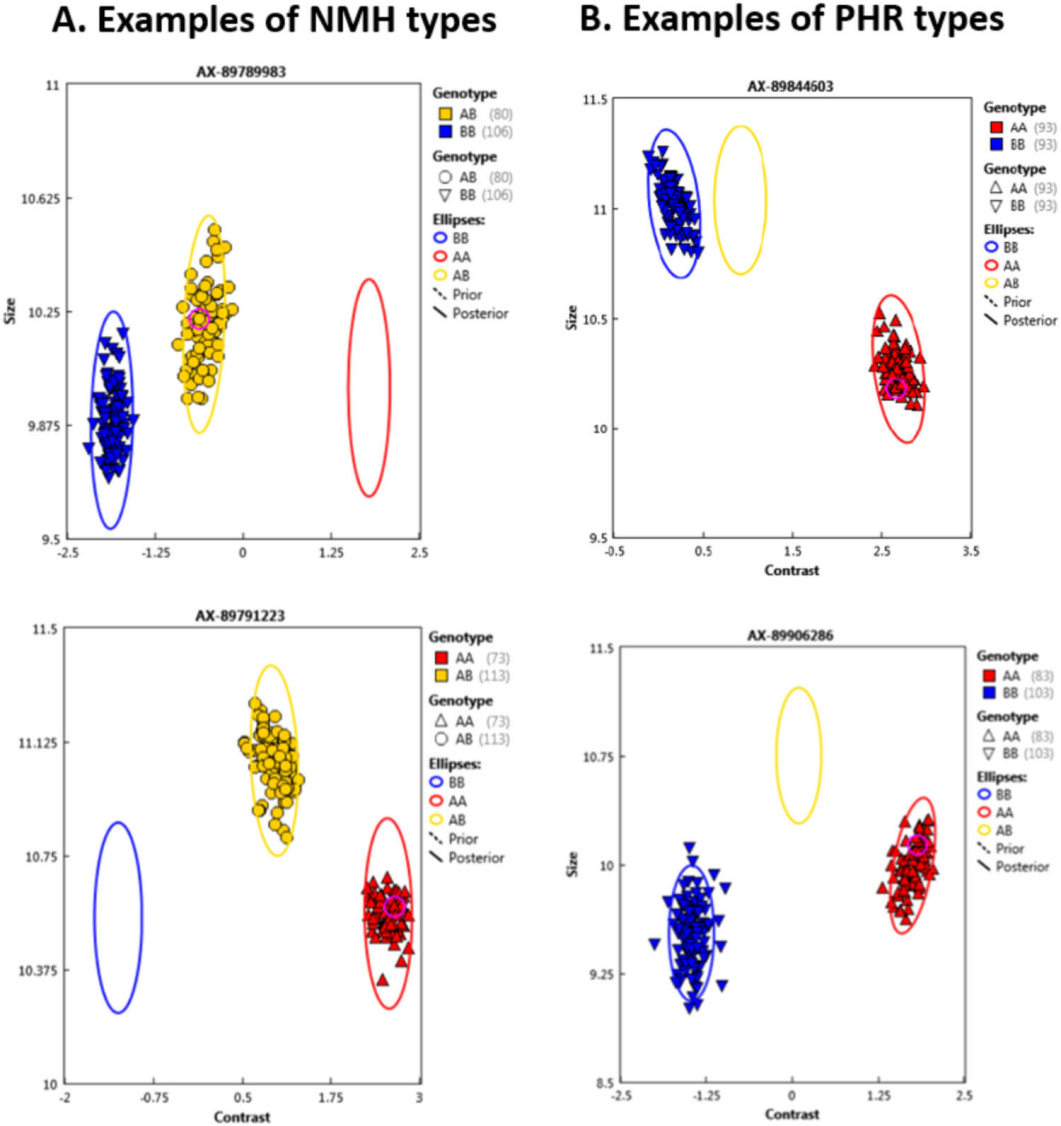
Examples of cluster diagrams of the (**a**) no minor homozygote (NMH) and (**b**) PolyHighResolution (PHR) types. Each diagram represents a different marker, and each colored dot (circle or triangle) represents an individual plant. For NMH marker AX-89789983 (**a**, top), plants with blue or yellow dots were coded by the Axiom Analysis Suite software as genotypes 2 or 1, respectively. For NMH marker AX- 89791223 (**a**, bottom), plants with yellow or red dots were coded as 1 or 0, respectively. For PHR markers AX-89844603 (**b**, top) and AX-89906286 (**b**, bottom), plants with blue or red dots were coded as 2 or 0, respectively

After genotyping on the IStraw90 array, all parental and 178 pentaploid plant samples yielded the data that passed the quality control criteria. The average call rate for passing samples was 99%. In total, 138,099 SNPs were called, and were classified into the six standard Affymetrix conversion types (performance categories) based on criteria displayed by their respective cluster patterns (e.g., Fig. 1) as explained elsewhere^21^.

The set of 16,074 markers belonging to the categories of NMH and PHR (Fig. 1) and defined as the best probe set by the Affymetrix Axiom Analysis Suite was selected. Of these, markers displaying matching genotype calls in L1 and BC6 were excluded from further consideration with the aim to insure marker segregation in the pentaploid population and to facilitate accurate assignment of alleles to their respective grandparental sources. Markers considered permissible for mapping were also required to display two-category segregation patterns in the pentaploid progeny population (Supplementary Table S1). Following application of all marker-selection criteria, 6586 markers remained for use in linkage mapping, and these markers were used as input for linkage analysis using JoinMap.

### Linkage analysis and map construction

At the LOD score of 8.0, linkage clusters containing <20 markers were eliminated from further consideration, leaving 29 clusters, the smallest of which (LG29) had 72 markers while all others had numbers of markers ranging from 118 to 368 (Table 1). In total, the 29 identified linkage groups comprised 6127 markers, of which 5274 were classified as NMH, and the rest (853) were classified as PHR, as listed in Supplementary Table S2. With respect to design categories^21^, 6049 of the mapped SNPs belonged to categories based on discovery in an octoploid germplasm panel, while 59 belonged to the F1D category based on discovery in diploid *Fragaria iinumae*, and 19 were so-called “codon based” SNPs. Among the 6049 “octoploid-based” markers^21^, 4620 were simple, di-allelic SNPs; 183 were multi-allelic “mSNPs”; 486 were indel (insertional or deletional) SNPs; and 760 belonged to one of the “haploSNP” categories (572 SNP–SNP, 64 indel–SNP, and 124 SNP-in-insertion).

**Table 1:**
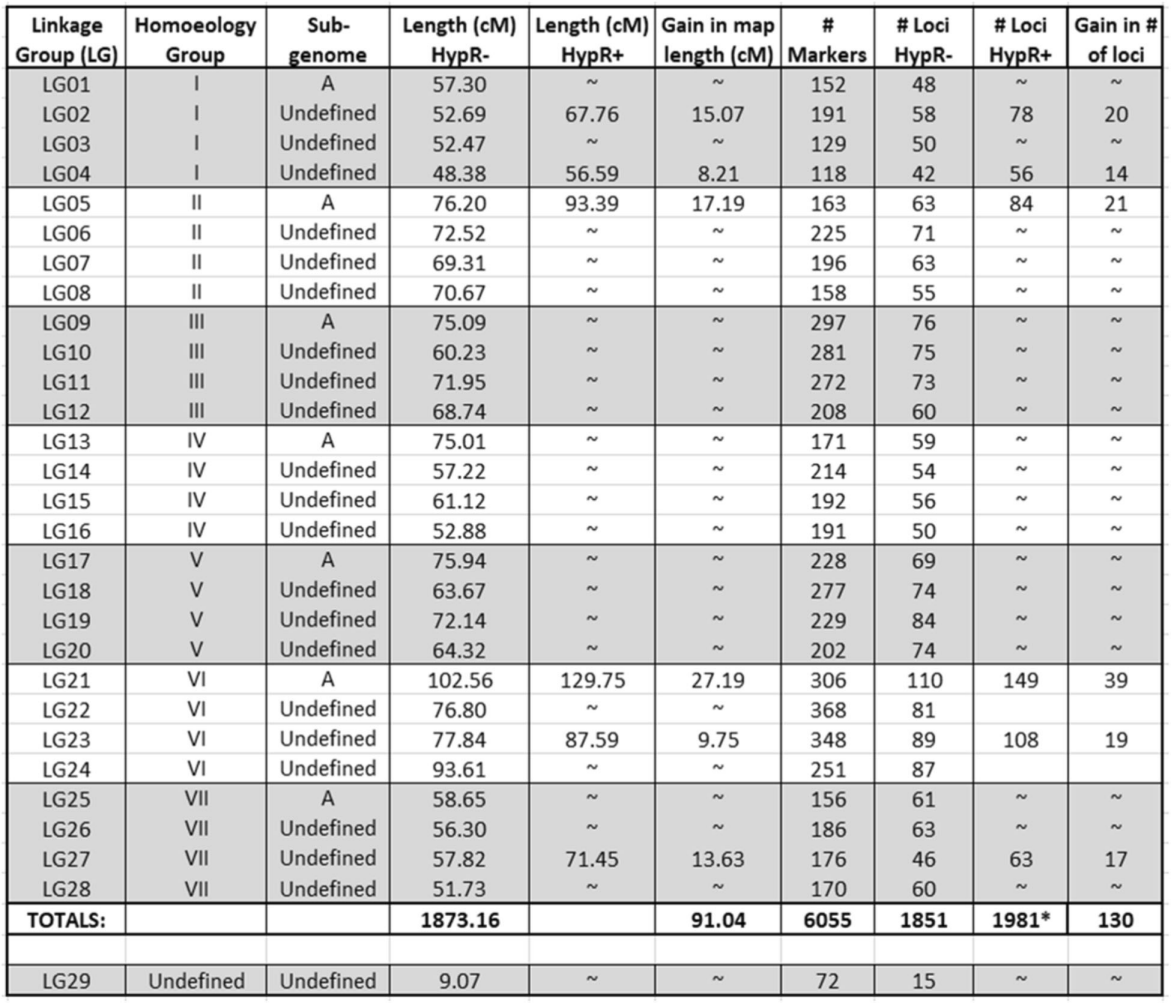
Map summary data

Parameters in columns with “HypR−" or “HypR+” in the headings (Length and # Loci headings) were calculated with the data from the respective HypR chromosome excluded (HypR−) or included (HypR+). *The total of 1981 at the bottom of the column headed “^#^Loci HypR+” is the sum over all 28 canonical LGs with the six HypR+ chromosomes included. The data for LG29 are included at the bottom of the table, but are not included in any of the totals

**Fig. 2:**
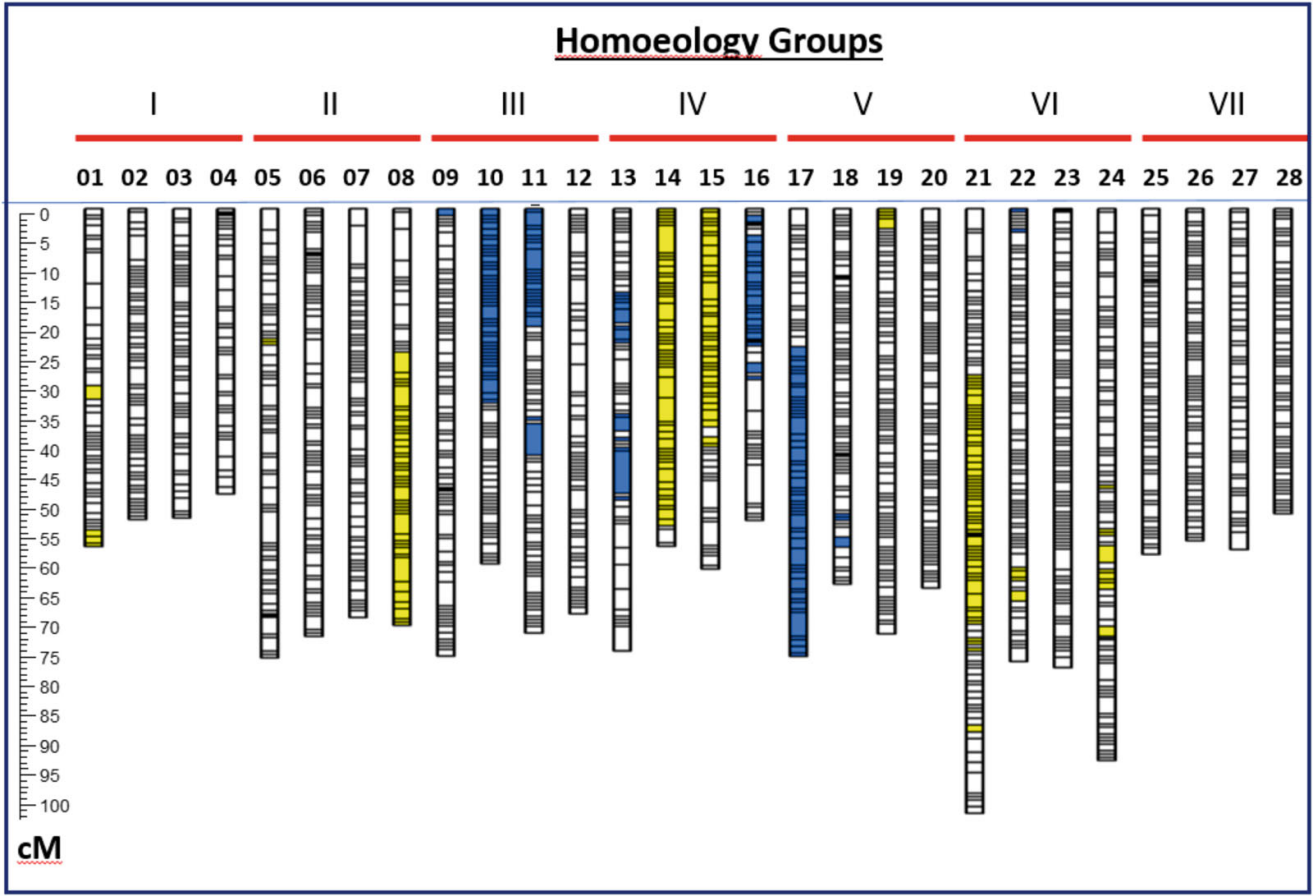
The canonical LB48 (*F. virginiana*) linkage map. This map was calculated after exclusion of the six HypR chromosomes from the data set. The color coding indicates approximate locations of regions of segregation distortion. Segregation bias favoring alleles from the maternal (L1) or paternal (BC6) parent of LB48 are indicated, respectively, by yellow or blue highlighting. The detailed presentation of marker segregation data and distortion is provided in Supplementary Table S2

Following construction of an initial map, the numbers of crossovers in each chromosome in each individual plant were counted, entailing examination of 5162 chromosomes (= 29 chromosomes per individual × 178 individuals). Surprisingly, it was found that six individuals each harbored one chromosome (a different chromosome in each case) exhibiting an abnormally high number of crossovers, as further detailed below. Due to the atypical nature of these six hyper-recombinant (HypR) chromosomes, they were provisionally excluded from the data set by converting their genotype calls to missing data. Singleton correction was then performed, and the map was recalculated, resulting in the canonical LB48 linkage map that is shown in Fig. 2, summarized in Table 1, and detailed in Supplementary Table S2. The effects on the map of restoring the six HypR chromosomes to the data set were subsequently examined, as described later.

### The canonical LB48 linkage map

For the purpose of defining a canonical (i.e., broadly representative) linkage map from *F. virginiana* LB48, we chose to exclude the data from the six HypR chromosomes, and to exclude LG29, which was anomalous not only for its low number of markers but in other ways as subsequently described. Thus, the canonical LB48 map (Fig. 2) contains 28 linkage groups (LGs), comprising 6055 array-based SNP markers of types detailed above. These markers are distributed over 1851 loci (marker bins), in linkage groups ranging in length from 48.38 cM to 102.56 cM, while excluded LG29 was only 9.07 cM in length (Table 1). The sum of the 28 canonical linkage group lengths is 1873 cM (Table 1). Over all 28 canonical linkage groups, the mean map distance between adjacent loci is 1.01 cM, and the mean marker density is 3.23 markers/cM.

### Linkage group numbering and subgenome assignment

Numbering of LGs was based on the following two criteria: assignment to homoeology groups and, where possible, subgenomic assignments. Linkage groups LG01 through LG28 were each unambiguously assigned to a homoeology group based on known marker locations^21^ on the seven pseudochromosomes (PCs) of the diploid Hawaii 4 Ver 1.1 reference genome^10^. Among these 28 LGs, best-percent matches ranged from 79% for LG02 up to 95% for LG06 (Table 2). The outcome of this procedure was that four linkage groups were assigned to each homoeology group. LG29 could not be unambiguously assigned; however, its best-percent match (39%) was to homoeology group V (Table 2, highlighted in yellow).

**Table 2:**
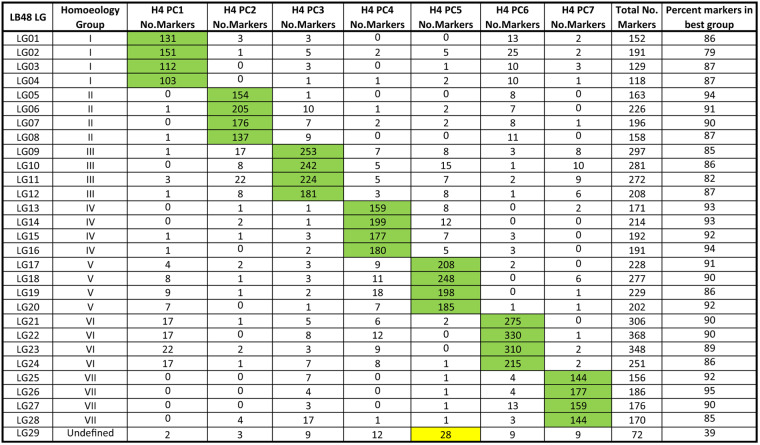
Assignment of linkage groups to homoeology groups

Based upon the employed procedure, linkage groups LG01 through LG28 were each assigned to a homoeology group corresponding to one of the seven pseudochromosomes (PCs) of the diploid Hawaii 4 (H4) Ver 1.1 reference genome^10^. Among these 28 LGs, the lowest percent match occurred in LG02, where 151 of 191 (=79%) mapped markers were located on H4 PC1. LG29 was not assigned to a homoeology group because its best percentage match (to H4 PC5) was only 39% (28 out of 72 matching markers)

Following the approach of Sargent et al.^19^, the 572 mapped markers of the haploSNP (SNP–SNP) design category (Supplementary Table S2) were used for the purpose of assigning LGs to subgenomic categories. The numbers of SNP–SNP markers on each chromosome assigned to the YN, YY, NY, and NN affinity categories is reported in Table 3. On the basis of these results, one member of each homoeology group could be unambiguously assigned to the “A” (*F. vesca*-derived) subgenome (Table 3). However, unambiguous assignment of LGs to an *F. iinumae*-derived (“B”) subgenome or any other subgenomic category was not achievable using the employed approach. Thus, the LG numbers LG01 through LG28 were assigned in ascending sets of four, such that the lowest LG number in each set was assigned to the subgenome A member of the respective homoeology group (Table 3), and the numbering order of the remaining three group members was arbitrary. Accordingly, the “A” subgenome in *F. virginiana* hybrid LB48 is represented by linkage groups LG01, LG05, LG09, LG13, LG17, LG21, and LG25 (Table 3), and no other subgenomes are defined.

**Table 3:**
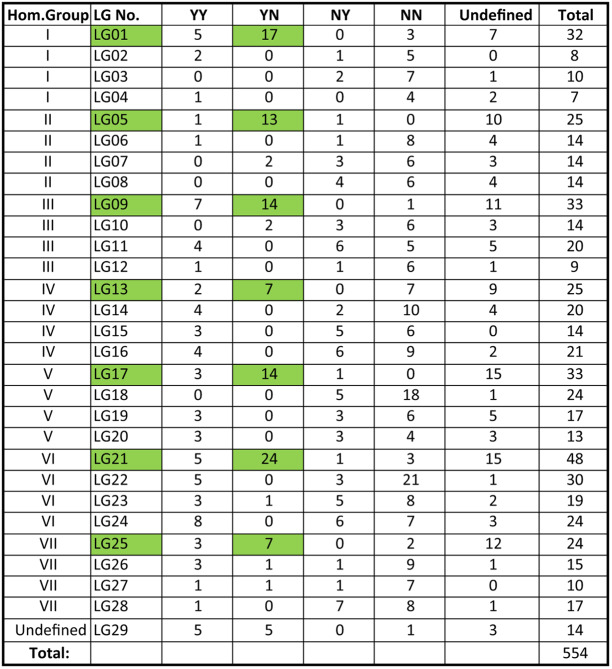
Subgenome assignments within homeology groups

The SNP–SNP marker-affinity method of Sargent et al.^19^ was applied as a means of assigning linkage groups to subgenomes. Linkage group (LG) affinities to the A subgenome of diploid *Fragaria vesca* are highlighted in green

### Comparison with DA × MO *F*. ×*ananassa* linkage map

**Fig. 3:**
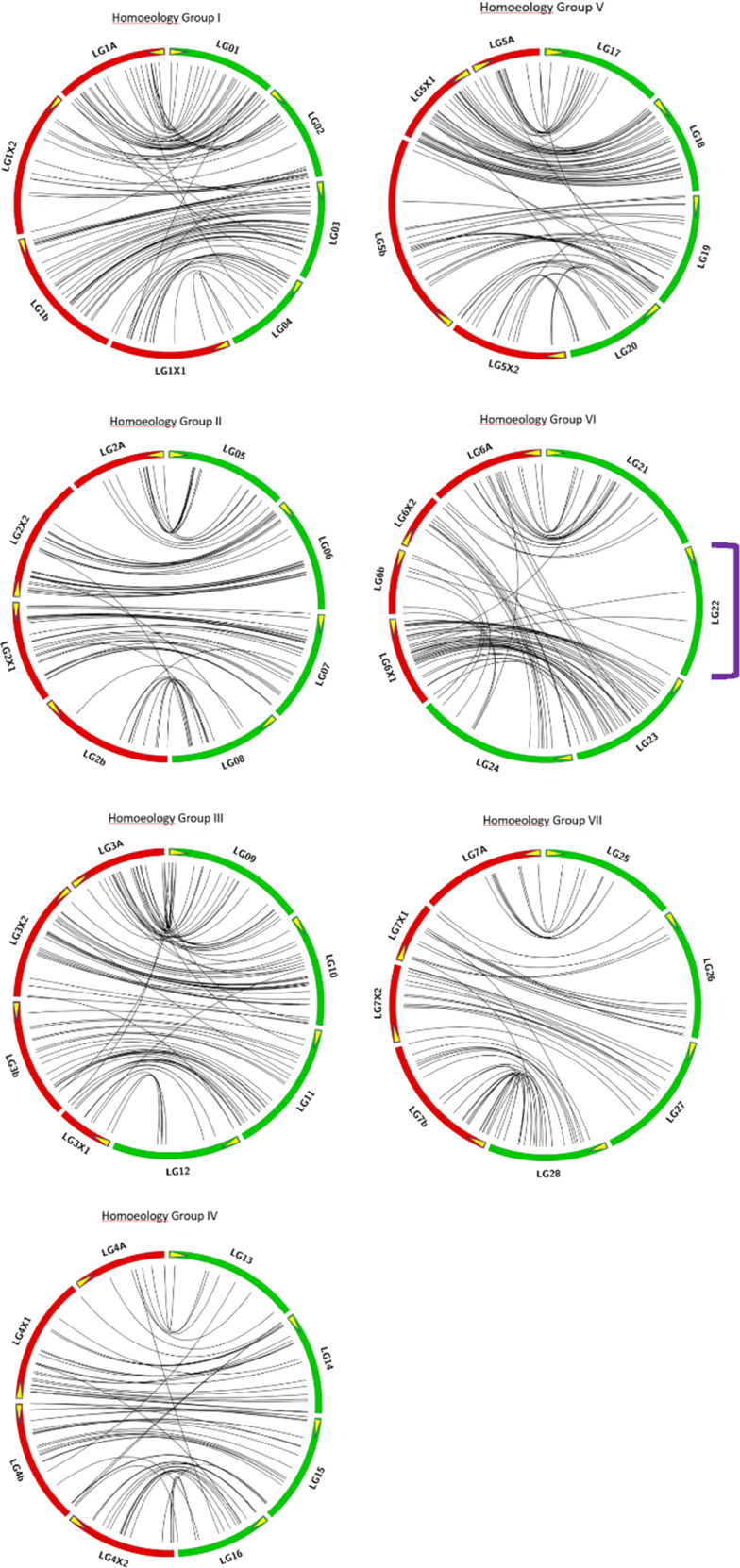
Synteny comparison of LB48 and DA × MO linkage maps. In these Circos diagrams, green and red arcs represent the linkage groups of the LB48 and DA×MO^19^ maps, respectively. Yellow arrows on the LG arcs indicate the start point (0cM end) of each LG. The purple bracket draws attention to LB48 linkage group LG22, which has no corresponding LG in the DA×MO map

When a direct comparison was made between the linkage group assignments of the 646 markers shared between the LB48 and DA × MO maps, it was found that the synteny of shared markers was 100% conserved: i.e., every one of the 646 shared markers was located in the same homology grouping in both maps. Within homoeology groups, synteny was also generally well conserved, (Fig. 3; Supplementary Table S3), with an interesting exception that in homoeology group VI, only three of the 368 markers on LB48 LG22 were shared with the DA × MO map, two of them linking to LG6X1, and one to LG6b (Fig. 3). Contrastingly, the markers on LB48 LG24 partitioned onto two different DA × MO LGs (Fig. 3) This comparative approach also identified two sets of seven LB48 linkage groups corresponding, respectively, to sets of LGs attributed to the *F. vesca* (A) and hypothesized *F. iinumae* (b) subgenomes specified in the DA × MO map (Fig. 3; Supplementary Table S3).

### Patterns of segregation distortion

Of the 6055 markers on the LB48 map, 1331 (22%) exhibited segregation distortion (X2 (1:1), *P* ≤ 0.05). At least one locus exhibited segregation distortion on 17 out of 28 canonical LGs (Fig. 2; Supplementary Table S2), including at least one member of every homoeology group except group VII, and all four members of homoeology group IV. Linkage group 22 displayed distortion favoring BC6 alleles at its top end, while distortion favored L1 alleles at its bottom end (Supplementary Table S2). Otherwise, eight linkage groups displayed regions of systematic distortion favoring alleles derived from maternal grandparent L1, including LG14 in which 200 out of 214 markers were distorted (Supplementary Table S2), while seven others displayed regions of systematic distortion favoring alleles derived from paternal grandparent BC6. Throughout the 28 canonical LGs, the most extreme distortion was seen in LG08, where the rare (BC6) allele frequency reached a low of 0.32 (or 57/178) (Supplementary Table S2). Even more extreme distortion was seen in LG29, in which every locus was highly distorted in favor of the BC6 allele, and the frequency of the rare (L1-derived) allele ranged among loci from 0.03 (or 5/178) to 0.05 (Supplementary Table S2).

### Crossover distribution, hyper-recombination (HypR), and interference

**Fig. 4:**
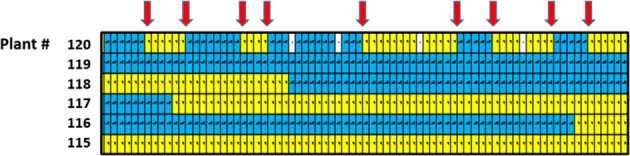
Visual display of crossover patterns, including a HypR chromosome. This image is abstracted (and rotated 90^o^ counterclockwise) from Supplementary Table S2, and shows genotypic data for part of LG04, with color-coded genotype calls for several individuals (rows), including for HypR chromosome LG04 in individual number 120. Each column reports the genotype calls (yellow=a; blue=b, white=missing data) for a different marker. The red arrows mark crossover points in individual #120

The marker segregation data for each linkage group is provided in Supplementary Table S2, where the location of each crossover point is indicated by a transition of marker color code from yellow (L1 = derived alleles) to blue (BC6-derived alleles), or vice versa (Fig. 4). The overall pattern of crossover distribution is summarized in Table 4. The total number of crossovers displayed by the data set (Supplementary Table S2) is 3429 (Table 4), and the mean number of crossovers per linkage group per individual is 0.66 (Table 4). Upon examination of these data, it was found that in each of six individuals, one chromosomal copy exhibited a markedly high number of crossovers (Fig. 4, Table 4; Supplementary Table S2): individual 12 had 27 crossovers in its copy of LG02; individual 14 had 48 crossovers in its copy of LG21; individual 94 had 19 crossovers in its copy of LG23; individual 114 had 24 crossovers in its copy of LG27; individual 120 had 15 crossovers in its copy of LG04; and individual 147 had 31 crossovers in its copy of LG05. Excluding these six instances of HypR, the mean number of crossovers per linkage group per individual was 0.63, with a range of from 0 to 4 excepting for one LG20 chromosome that had seven crossovers in individual 141 (Table 4; Supplementary Table S2).

**Table 4:**
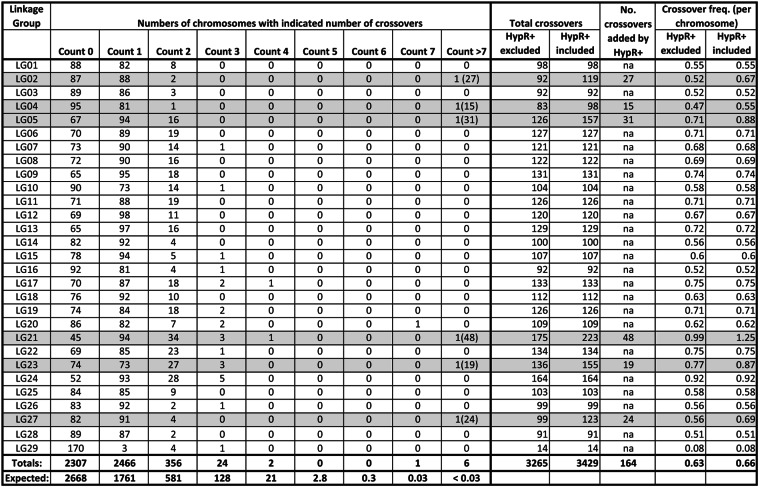
Crossover counts and distributions

For each linkage group, the indicated “Counts” are the numbers of individual chromosomes out of a possible 178 (i.e., total number of progeny individuals) with the indicated number of crossovers. For instance, for LG02 there were 87 chromosomes with no crossovers, 88 chromosomes with one crossover, two chromosomes with two crossovers, and one chromosome with 27 crossovers. As an example of obtaining total crossover numbers: for LG02, the total number of crossovers, including those in the HypR chromosome is given by: (1 × 88) + (2 × 2) + (1 × 27) = 119. Crossover frequency per linkage group is calculated as the total number of crossovers for that LG divided by either 177 (if the HypR chromosome is excluded) or 178 (if the HypR chromosome is included). The “expected” numbers in the bottom row are based on a Poisson-based analysis as described in the text

The distribution of chromosomes exhibiting 0, 1, 2, 3, or more crossovers is expected to follow a Poisson distribution^28^, where “m” is the mean event frequency, and “i” is the specific crossover count, in the present case ranging from 0 to 7. The Poisson frequency distribution was calculated as:

$${\mathrm{Expected}}\,{\mathrm{Freq}}\,\left( {\mathrm{i}} \right) = \left( {{\mathrm{e}}^{ - {\mathrm{m}}}{\mathrm{m}}^{\mathrm{i}}} \right)/{\mathrm{i}}!$$

Using the mean observed frequency (m) of 0.66 crossover points per chromosome (Table 4), as determined with inclusion of the six HypR chromosomes, the Poisson equation yielded the expectations listed in the bottom row of Table 4. A chi-square goodness-of-fit test was then used to compare the observed and expected numbers of chromosomes exhibiting the indicated numbers of crossovers (0, 1, 2, 3, 4, 5, 6, 7), and the observed values differed highly significantly from the expectations (X^2^ = 554, *P* < 0.001, df = 7). Specifically, the observed numbers of chromosomes with two or more crossovers were less than the expected numbers for all crossover count categories from 2 to 7 (Table 4), and also for the 0 crossover category. In contrast, 2466 chromosomes had a single crossover, substantially exceeding the expected number of 1761 for chromosomes with a crossover count of 1.

**Fig. 5:**
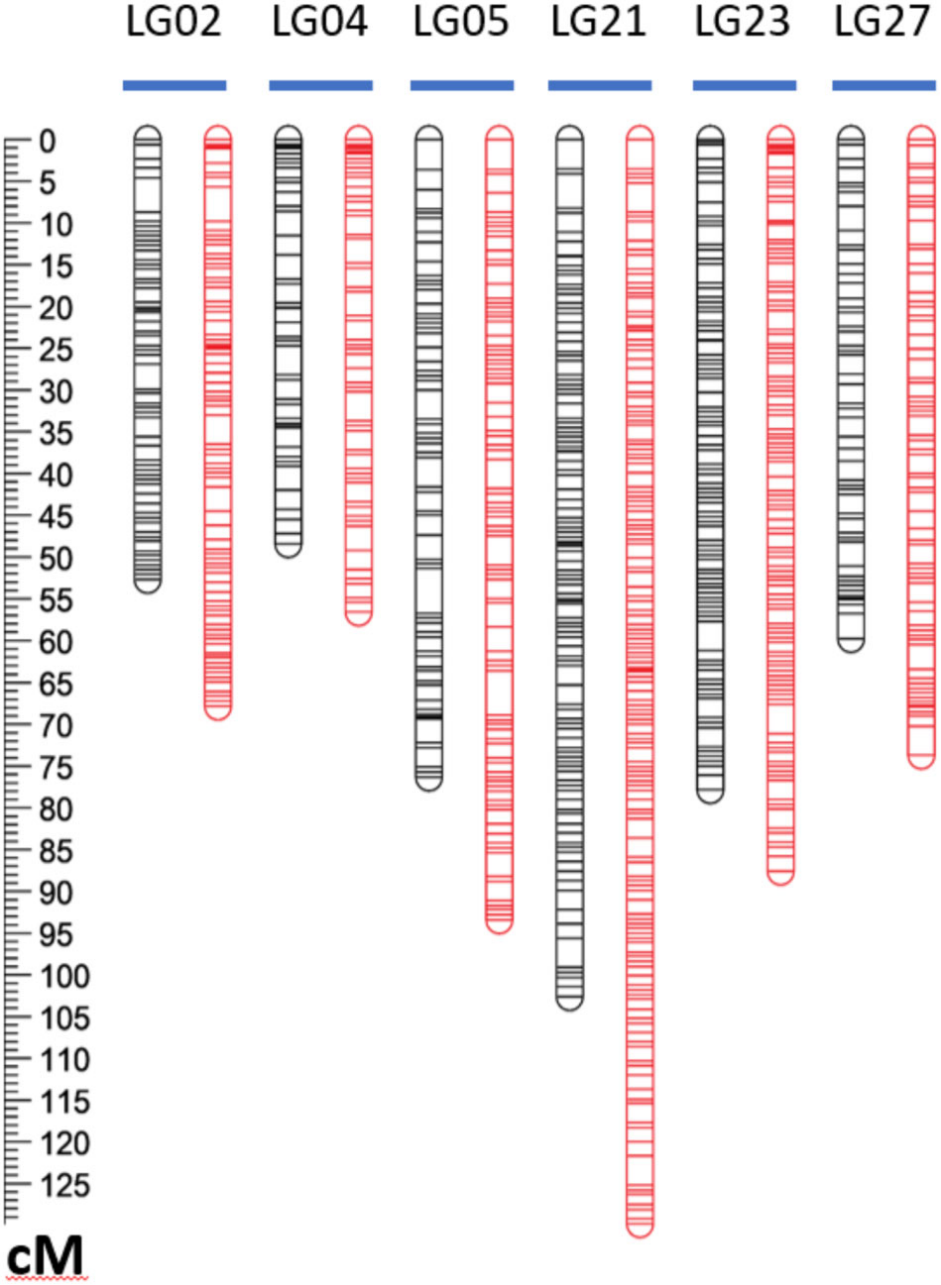
Comparisons of six linkage groups with and without hyper-recombination. For each linkage group pair, the version on the left (black) is calculated with the segregation data from the HypR chromosome excluded, as done in the canonical map (Fig. 2), while the version on the right (red) is calculated with the segregation data from the HypR chromosome included

In total, 164 crossovers, or 4.8% of the total number of 3429 crossovers, occurred in the six exceptional HypR chromosomes, which comprised just 0.1% of the 5162 individual chromosomes in the total data set. Due to the atypically high level of crossing over in these six chromosomes, their respective marker data were excluded (by treating as missing data) from the calculation of the canonical LB48 linkage map, as previously described (Fig. 2). The effect of including the HypR-related data was then examined by recalculating the six respective linkage groups after restoring the HypR-related data (Fig. 5, Tables 1 and 4; Supplementary Table S2).

Inclusion of the segregation data from the six HypR chromosomes added a combined total of 91.04 cM to the six linkage groups in question, and increased their combined number of loci by 130 (Table 1, Supplementary Table S2, and Fig. 5). The increase in locus number upon inclusion of HypR-related data resulted from the splitting of affected loci that contained multiple markers (see boxed LG regions in Columns C, D, and E of Supplementary Table S2).

## Discussion

Here, we report the use of a pentaploid (2n = 5x = 35) progeny population for the purpose of constructing a high-resolution linkage map, the canonical LB48 map, for the ancestral octoploid (2n = 8x = 56) strawberry species *F. virginiana*. The LB48 linkage map comprises 28 linkage groups distributed into seven homoeology groups of four members each, matching the expectations for a linkage map of a species with haploid chromosome number of 1n = 4x = 28, as is the case for *Fragaria virginiana*.

### Map resolution

The LB48 map, comprising 6055 SNP markers distributed over 1851 loci and 1873 cM of total map length, is the first high-resolution linkage map of the ancestral octoploid species *F. virginiana*. Based as it is on segregation data from 178 gametes derived from parent LB48, the shortest theoretical distance between adjacent loci in the map is 0.56 cM (= 1/178), as compared with the observed mean distance of 1.01 cM. Any intervals shorter than the theoretical minimum, a few instances of which are evident in the “Positions” columns of Supplementary Table S2, are the artifactual consequences of an interval splitting convention in JoinMap that is used when a precise crossover location cannot be assigned due to missing data at the crossover point. The mapped markers are generally well-distributed, with the largest interval between adjacent loci of 7.24 cM being located near the middle of LG13 (Fig. 2; Supplementary Table S2).

Within each homoeology group, the lowest LG number in an ascending series was assigned to the linkage group identified as corresponding to the “A” subgenome (Table 3), which is widely accepted to be derived from its ancestral diploid (2n = 2x = 14, AA) *Fragaria vesca*^19,30^. The approach to subgenome identification taken here was patterned after that of Sargent et al.^19^, who defined the “A” subgenome linkage groups of the *F*. ×*ananassa* DA × MO linkage map based upon the affinities of IStraw90 haploSNP (SNP–SNP) marker tag sequences to the corresponding sites in the *F. vesca* “Hawaii 4” Ver 1.1 reference genome. Similarly, these investigators^19^ tentatively identified a second *F*. ×*ananassa* subgenome, designated “b”, that was putatively derived from the ancestral B genome diploid (2n = 2x = 14, BB) *Fragaria iinumae*. This assignment was achieved by utilizing *F. iinumae* Illumina reads aligned to the corresponding SNP target sites in the Hawaii 4 Ver 1.1 genome assembly. In our present study, only the A-subgenome linkage groups of the LB48 map could be confidently identified based upon SNP–SNP marker tag affinities.

### Comparison with the *F*. ×*ananassa* DA × MO linkage map

Several insights were gained via comparison of the LB48 map to the previously published *F*. ×*ananassa* DA × MO linkage map^19^, both of which were based on SNP markers genotyped on the IStraw90 array. First, all 646 shared markers had the same homoeology group memberships in the LB48 map as they did in the DA × MO map, while the extents of synteny conservation and collinearity among members of each homoeology group was variable, but generally good. Interestingly, a clear correspondence was evident between each LB48 linkage group and one respective DA × MO linkage group, with two exception. In LG22, the LB48 linkage group with the most markers on the LB48 map, shared only three markers with the entire DA × MO map. Thus, LG22 has no counterpart on the DA × MO map. In contrast, LG24 displays correspondence at its “zero” end to DA × MO LG6X2, and at its opposite end to DA × MO LG6b (Fig. 3). This finding, which is suggestive of homoeologous substitution, supports the hypothesis^19^ that subgenome composition may be variable among octoploid strawberry species and even perhaps within species.

At the level of subgenome composition, the synteny comparison agreed with the previously discussed haploSNP analysis of the LB48 map in its identification of seven LGs corresponding to those comprising the A subgenome set in the DA × MO map. In addition, the synteny comparison identified correspondences with the seven DA × MO linkage groups provisionally identified as comprising a “b” subgenome derived from ancestral diploid *F. iinumae*. The seven LB48 LGs in question are LGs 03, 08, 11, 15, 19, 24, and 28. Their respective correspondences to DA × MO linkage groups are reported in Supplementary Table S3, and are displayed in Fig. 3. However, we have concluded that to define these seven LGs as comprising an *F. iinumae* subgenome in *F. virginiana* on the basis of the DA × MO correspondence would be premature, given that the “b” subgenome designations assigned in the DA × MO map were considered as provisional by those investigators, who emphasized that the supporting haploSNP-affinity data for this subgenomic assignments were not highly robust^19^.

### LG29

The excluded linkage group LG29 was not assigned to a homoeology group, and was atypical in several ways. It had only 72 markers as compared with the mean of 216 markers for the 28 canonical linkage groups, its map length of 9.07 cM was much shorter than the canonical mean of 66.89 cM, and it exhibited extreme segregation distortion. Nevertheless, no evidence of genotype miss-calling was found via careful examination of the cluster diagrams for the 72 markers in LG29. Thus, although we have chosen to exclude LG29 from the canonical LB48 map, its existence is reported and carefully documented here to allow for the possibility that a biological explanation for its anomalous characteristics will emerge from follow-up studies.

### Segregation distortion

Significant deviation (*P* ≤ 0.05) from the expected 1:1 segregation ratio was seen in 1331 markers, or 22% of the 6055 markers comprising the LB48 map. Systematic bias toward alleles derived from grandparents L1 or BC6 was seen in parts of eight linkage groups for each, while in LG22 the segregation bias favored BC6 alleles at the top of the linkage group and L1 alleles at the bottom. Although it has been shown that the presence of segregation distortion is not an impediment to accurate map construction^31^, some investigators have chosen to exclude markers exhibiting segregation distortion from use in map construction^22,32^. If we had excluded markers exhibiting segregation distortion from our map construction pipeline, substantial regions of the map would have been lost, including: the top 32 cM of LG10; the bottom 46 cM of LG08; 52 cM of LG17; and all but 3 cM of LG14; as well as the entirety of LG29. Particularly when working with undomesticated germplasm and/or wide crosses, the automatic exclusion of markers exhibiting segregation distortion seems inadvisable, given the risk that genomic regions containing genes influencing gametophytic and/or zygotic viability or competitiveness may not be represented in the resulting map.

### The distribution of crossovers: evidence for interference

In our data set (Table 4), the mean number of crossover points per chromosome was 0.66 or 0.63, respectively, depending on whether the crossover counts from the six HypR chromosomes were or were not included in the calculation. Given this information, a set of expectations for the categorical distribution of numbers of crossover points per chromosome could be calculated. The Poisson distribution is an appropriate mathematical tool for describing crossover frequencies^28^. The outcome of our Poisson analysis led to two notable conclusions. First, the lower than expected crossover counts in each of the multiple (two or more) crossover categories is indicative of interference, whereby the presence of one crossover tends to suppress the occurrence of additional crossovers involving the same chromosome. However, the lower than expected count for the 0 crossover category and higher than expected count for the 1 crossover category is suggestive of a mechanism promoting the occurrence of a crossover. Thus, the overall pattern of disagreement between observed and expected crossover counts implies the existence of interacting mechanisms suppressing and promoting crossing over.

### Hyper-recombination and its possible utility

Turning now to the HypR chromosomes, the Poisson analysis indicated that the occurrence of even one chromosome with 15 or more crossovers was vanishingly small, and that therefore the occurrence of six such chromosomes, with crossover numbers ranging from 15 to 48, cannot be attributed to chance. Importantly, the observed hyperelevation of recombination in just six out of 5162 chromosomes assayed is not incremental or genome-wide, but instead is drastic and narrowly focused. Thus, any proposed mechanism must offer an explanation not only for a hyperelevation in crossover number, but also its narrow yet seemingly arbitrary focus limited to one chromosome in each affected individual.

If the causal factors underlying the observed instances of hyper-recombination can be identified, they may be amenable to manipulation to the benefit of plant breeders and geneticists. Inclusion of the HypR chromosome in linkage calculations in each case lengthened the respective linkage group, and added additional map loci. The latter resulted from the splitting of individual marker bins (see Supplementary Table S2) into two or more bins (map loci), and thus added resolution to the map. Such added linkage map resolution would benefit investigations of gene/marker/trait associations, and would add resolution to the anchoring of genome assemblies.

The manipulation of crossover frequency and/or distribution has gained recent attention as a potential means of accelerating the progress of plant breeding programs^33,34^. One promising approach has been the genetic manipulation of genes encoding functions involved in meiotic crossing over^35^. Another is the manipulation of environmental factors, such as temperature^36^. Such manipulations have offered promise of increasing crossover frequencies on a genome-wide scale, but have not, to our knowledge, resulted in the phenomenon of episodic, chromosomally focused leaps in recombination frequency that we have described. Accordingly, we hypothesize that the causal molecular mechanisms underlying hyper-recombination in our study system may be distinct from those currently being subjected to experimental manipulation. One avenue toward understanding this phenomenon could be the genomic resequencing of the six affected LB48 progeny plants, with the aim of identifying sequence features that might be common to the sites of crossing over in HypR chromosomes. A second would be to acquire a broader sampling of HypR individuals for study. Although currently without causal explanation, our results point to the episodic induction of hyper-recombination, if it can be successfully manipulated, as a new and potentially quite valuable addition to the plant breeding/genomics toolkit.

### Merits of a pentaploid progeny population for linkage mapping in octoploid *Fragaria*

The LB48 map is the first linkage map in octoploid *Fragaria* to be based on segregation analysis of a pentaploid progeny population. In effect, these pentaploid progeny plants were allohaploids, each carrying one haploid complement (4x = 28) of chromosomes from the LB48 paternal parent, and one haploid complement (1x = 7) from the *F. vesca* “Hawaii 4” maternal parent. Despite the reported level of heterozygosity in Hawaii 4^37^, our reliance upon polymorphisms between the paternal grandparents L1 and BC6 and the exclusion of markers exhibiting three-category (aa:ab:bb) segregation patterns sufficed to exclude any markers influenced by heterozygosity in Hawaii 4 from the final map. Accordingly, the LB48 map is a uniparental map, as distinct from most if not all octoploid *Fragaria* maps reported to date, which have been based on pseudo-testcrosses between two heterozygous parents, necessitating the initial construction and deconvolution of separate maternal and paternal maps.

The use of a pentaploid mapping population offers both advantages and disadvantages for linkage map construction in octoploid *Fragaria*. Marker genotype assignment is simplified in the pentaploid population, because all marker segregation patterns are of the two-category type (“a” versus “b”), and as noted above all segregation derives from the heterozygosity of just one parent. A benefit of two-category segregation is the simplification of the cluster analysis performed by the Affymetrix Axiom Analysis Suite software. A challenge associated with SNP genotyping in a polyploid is cluster compression, which arises because the dosage of the marker (or “minor”) allele, present in 0, 1, or 2 copies, must be ascertained against a background of a larger and variable number of “major allele” copies present in homoeologous chromosomes but not segregating^21^. The separation of genotype calls into just two clusters for PHR as well as NMH marker types (Fig. 1) rather than three clusters for PHR markers as seen in pseudo-testcross progenies reduces the impact of the cluster compression that is associated with polyploidy in the Affymetrix system^21^, thus enhancing the robustness of genotype calling. Cluster compression is also reduced in the pentaploid as compared with an octoploid setting because, for a given marker, signal is coming from at most five homologs instead of up to eight.

The effective haploidy of the pentaploid progeny offers maximum clarity for purposes of dissecting complex patterns of multiple crossing over. However, with respect to the cost of genetic information, the pentaploid approach is more expensive, in that each progeny individual represents just one informative, octoploid-derived gamete, while in the pseudo-testcross context each progeny individual is constituted of two informative gametes, thus doubling the amount of genetic information available per unit cost. Finally, for purposes of marker/trait association analysis, pentaploid populations have the disadvantage of sexual sterility, thus precluding identification of marker associations with traits involving fruit and seed production.

### Effectiveness of the IStraw90 SNP array in *F. virginiana*

Both the *F. virginiana* LB48 and *F*. ×*ananassa* DA × MO^19^ maps were constructed from SNP markers genotyped on the IStraw90 Strawberry SNP Array. Comparison of these maps provides insight into the performance of the IStraw90 array in *F. virginiana*, a strawberry species for which the array was not specifically designed. The LB48 and DA × MO maps are, respectively, similar in overall length (1873 versus 1820 cM), while the LB48 map has fewer markers (6055 versus 8407) and a proportionately lower locus density (0.99 versus 1.3 loci per cM). The IStraw90 array is populated primarily by markers discovered in an *F*. ×*ananassa* germplasm panel^21^; however, the substantial number of markers available in our *F. virginiana* cross indicates that the IStraw90 array is also an effective genotyping platform in this ancestral octoploid species. For future studies of *F. virginiana*, it is noteworthy that only 2427 (or 39.6%) of the 6127 LB48 markers (including those on LG29) are represented on the derivative Axiom IStraw35 array^38^, whereas 4583 (or 54.5%) of the 8407 DA × MO markers are represented there. Thus, for future studies of marker/gene associations with traits of interest in *F. virginiana*, the IStraw90 array will likely be the preferred genotyping platform.

## Conclusion

In conclusion, the canonical LB48 linkage map provides a useful new genomic resource for the *Fragaria* research community. The LB48 map provides a new genomic resource for identifying marker–trait associations and anchoring genome assemblies in *F. virginiana*, and by extension for the octoploid cultivated strawberry *F.* ×*ananassa*. The features and properties of the LB48 map also provide insight into the consequences of conducting linkage analysis in a pentaploid mapping population, and into the performance of the IStraw90 strawberry SNP array as a basis for linkage mapping in *F. virginiana*, a strawberry species other than those for which the IStraw90 array^21^ and its derivative IStraw35 array^38^ were designed.

Finally, our data have unexpectedly revealed the existence of an intriguing and potentially useful genetic phenomenon, meiotic hyper-recombination (HypR). To our knowledge, this phenomenon has not previously been described in plant linkage mapping studies. However, the possibility may be considered that some previously published data sets may provide evidence of hyper-recombination in the absence of its recognition by the respective investigators. Alternately, just as some investigators have excluded markers with distorted segregation ratios from mapping data sets, some investigators may have discarded data from individuals exhibiting hyper-recombination or other departures from normal expectations. It will be of considerable interest to see whether our report encourages the recognition and reporting of other instances of hyper-recombination in plants and other eukaryotes.

## Supplementary information

Supplementary Table S1 - Code Conversion Rubric

Supplementary Table S2 - Table Heading - Mapping Data

Supplementary Table S2 - Mapping Data Spreadsheet

Supplementary Table S3 - Map Comparison

## Data Availability

All data associated with this study will be publicly available. The complete set of genotype calls upon which the map is based is provided in Supplementary Table S2. The locus identifiers in that table refer to SNP locus information that was previously published^21^.

## References

[1] Marx V (2013). The genome jigsaw. Nature.

[2] Kyriakidou M, Tai HH, Anglin NL, Ellis D, Strömvik MV (2018). Current strategies of polyploid plant genome sequence assembly. Front. Plant Sci..

[3] Mascher M, Stein N (2014). Genetic anchoring of whole-genome shotgun assemblies. Front. Genet..

[4] Chapman JA (2015). A whole-genome shotgun approach for assembling and anchoring the hexaploid bread wheat genome. Genome Biol..

[5] Verde I (2013). The high-quality draft genome of peach (*Prunus persica*) identifies unique patterns of genetic diversity, domestication and genome evolution. Nat. Genet..

[6] Neale DB (2014). Decoding the massive genome of loblolly pine using haploid DNA and novel assembly strategies. Genome Biol..

[7] Shaw D (1997). Trait mean depression for second-generation inbred strawberry populations with and without parent selection. Theor. Appl. Genet..

[8] Liu B, Poulsen EG, Davis TM (2016). Insight into octoploid strawberry (*Fragaria*) subgenome composition revealed by GISH analysis of pentaploid hybrids. Genome.

[9] Shulaev V (2011). The genome of woodland strawberry (*Fragaria vesca*). Nat. Genet..

[10] Sargent DJ (2011). Simple sequence repeat marker development and mapping targeted to previously unmapped regions of the strawberry genome sequence. Plant Genome.

[11] Rousseau-Gueutin M (2008). Comparative genetic mapping between octoploid and diploid Fragaria species reveals a high level of colinearity between their genomes and the essentially disomic behavior of the cultivated octoploid strawberry. Genetics.

[12] Weebadde C (2008). Using a linkage mapping approach to identify QTL for day‐neutrality in the octoploid strawberry. Plant Breed..

[13] Zorrilla-Fontanesi Y (2011). Theor. Appl. Genet..

[14] Sargent DJ (2012). Theor. Appl. Genet..

[15] van Dijk T (2014). Genomic rearrangements and signatures of breeding in the allo-octoploid strawberry as revealed through an allele dose based SSR linkage map. BMC Plant Biol..

[16] Lerceteau-Köhler E (2012). Genetic dissection of fruit quality traits in the octoploid cultivated strawberry highlights the role of homoeo-QTL in their control. Theor. Appl. Genet..

[17] Isobe SN (2013). DNA Res..

[18] Tennessen JA, Govindarajulu R, Ashman T-L, Liston A (2014). Evolutionary origins and dynamics of octoploid strawberry subgenomes revealed by dense targeted capture linkage maps. Genome Biol. Evolution.

[19] Sargent D (2016). Plant Sci..

[20] Gezan SA, Osorio LF, Verma S, Whitaker VM (2017). An experimental validation of genomic selection in octoploid strawberry. Hortic. Res..

[21] Bassil NV, Davis TM (2015). BMC Genomics.

[22] Hossain MR (2019). Sci. Rep..

[23] Lee YR, Lee J (2017). Korean J. Breeding Sci..

[24] Spigler R, Lewers K, Main D, Ashman T (2008). Genetic mapping of sex determination in a wild strawberry, *Fragaria virginiana*, reveals earliest form of sex chromosome. Heredity.

[25] Goldberg MT, Spigler RB, Ashman T-L (2010). Comparative genetic mapping points to different sex chromosomes in sibling species of wild strawberry (*Fragaria*). Genetics.

[26] Mahoney LL (2016). A high-density linkage map of the ancestral diploid strawberry, *Fragaria iinumae*, constructed with single nucleotide polymorphism markers from the IStraw90 array and genotyping by sequencing. The Plant Genome.

[27] Galbraith DW (2009). Simultaneous flow cytometric quantification of plant nuclear DNA contents over the full range of described angiosperm 2C values. Cytom. Part A: J. Int. Soc. Advancement Cytom..

[28] Griffiths, A. J. et al. *An Introduction to Genetic Analysis* (Macmillan, 2005).

[29] Krzywinski MI (2009). Circos: an information aesthetic for comparative genomics. Genome Res..

[30] Yang Y, Davis TM (2017). A new perspective on polyploid *Fragaria* (strawberry) genome composition based on large-scale, multi-locus phylogenetic analysis. Genome Biol. Evolution.

[31] Hackett C, Broadfoot L (2003). Effects of genotyping errors, missing values and segregation distortion in molecular marker data on the construction of linkage maps. Heredity.

[32] Li Y, He M (2014). Genetic mapping and QTL analysis of growth-related traits in *Pinctada fucata* using restriction-site associated DNA sequencing. PLoS ONE.

[33] Serra H (2018). Massive crossover elevation via combination of HEI10 and recq4a recq4b during *Arabidopsis* meiosis. Proc. Natl Acad. Sci. USA.

[34] Blary A, Jenczewski E (2019). Manipulation of crossover frequency and distribution for plant breeding. Theor. Appl. Genet..

[35] Fernandes JB, Séguéla-Arnaud M, Larchevêque C, Lloyd AH, Mercier R (2018). Unleashing meiotic crossovers in hybrid plants. Proc. Natl Acad. Sci. USA.

[36] Modliszewski JL (2018). Elevated temperature increases meiotic crossover frequency via the interfering (Type I) pathway in *Arabidopsis thaliana*. PLoS Genet..

[37] Hawkins C, Caruana J, Schiksnis E, Liu Z (2016). Genome-scale DNA variant analysis and functional validation of a SNP underlying yellow fruit color in wild strawberry. Sci. Rep..

[38] Verma, S. et al. Development and evaluation of the Axiom® IStraw35 384HT array for the allo-octoploid cultivated strawberry *Fragaria* ×*ananassa*. In 8th International Strawberry Symposium (Vol. 1156, pp. 75−81). (Acta Horticulturae; Vol. 1156). International Society for Horticultural Science.

