# Supplementary material for: A pentaploid-based linkage map of the ancestral octoploid strawberry Fragaria virginiana reveals instances of sporadic hyper-recombination
Source: Hortic Res. 2020 May 7;7:77. doi: 10.1038/s41438-020-0308-2 (PMC7206004; doi:10.1038/s41438-020-0308-2)

**Supplementary Table S1. Rubric for conversion of genotyping codes from Affymetrix (A) to JoinMap (JM) format.**  For each table row, columns 1 and 2 display a permissible combination of paternal grandparental (L1 and BC6) genotypes, the “5x codes” column displays a permissible pair of pentaploid (5x) codes, and the “A -> JM” columns display the respective code conversion formulas. For instance, in the first row, when L1 and BC6 codes were 0 and 1, respectively, and the pentaploid plants were coded either 0 or 1, then the marker qualified for mapping, in which case the pentaploids with Affymetrix (A) codes of 0 and 1 would be assigned JoinMap (JM) codes of “a” and “b”, respectively.


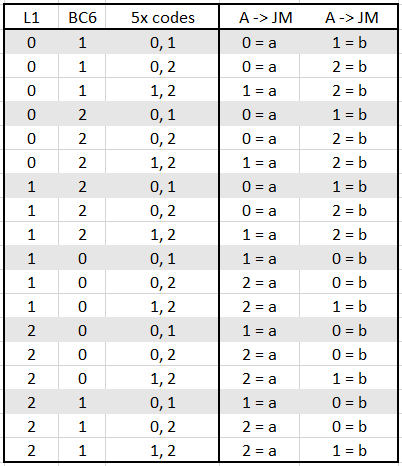

Supplement: Supplementary file 1 — Supplementary Table S1 - Code Conversion Rubric [file 41438_2020_308_MOESM1_ESM.docx]
