# Supplementary material for: A pentaploid-based linkage map of the ancestral octoploid strawberry Fragaria virginiana reveals instances of sporadic hyper-recombination
Source: Hortic Res. 2020 May 7;7:77. doi: 10.1038/s41438-020-0308-2 (PMC7206004; doi:10.1038/s41438-020-0308-2)
Supplement: Supplementary file 2 — Supplementary Table S2 - Table Heading - Mapping Data [file 41438_2020_308_MOESM2_ESM.docx]

**Supplementary Table S2. Mapping data and genotype calls.** Column A is a tracking column that can be used to restore the default row order after any rearrangement of rows by sorting. Column B lists the linkage group (LG) numbers in order from LG01 to LG29. Column C (Locus ID) lists the marker ID numbers as originally assigned by Bassil and Davis et al. (2015). Column D lists the cM positions of the 1,866 loci in the Canonical LB48 map (i.e., the map version calculated after exclusion of marker data from the six HypR chromosomes). Column E lists the loci and locus positons in the six LGs affected by hyper-recombination after restoration of the data from the HypR chromosomes to the data set. Boxes around sets of makers in columns C, D, and E draw attention to LG regions where loci were split by inclusion of HypR data, thereby increasing map resolution. Columns F, G, and H provide the allele (a, b) or missing data (-) counts for each marker. Columns I, J, K report on the X^2^ parameters and calculations related to the allele counts. Column L reports the performance category (PolyHighResolution = PHR, or NoMinorHomozygote = NMH) for each marker, while Column M reports the respective marker design categories as specified in Bassil and Davis et al. (2015. The genotype calls for each of the 178 pentaploid progeny individuals and each of the 6,127 mapped markers are provided in columns N through GI.

**See separate Excel spreadsheet for Table S2 data.**
