# Supplementary material for: A pentaploid-based linkage map of the ancestral octoploid strawberry Fragaria virginiana reveals instances of sporadic hyper-recombination
Source: Hortic Res. 2020 May 7;7:77. doi: 10.1038/s41438-020-0308-2 (PMC7206004; doi:10.1038/s41438-020-0308-2)
Supplement: Supplementary file 4 — Supplementary Table S3 - Map Comparison [file 41438_2020_308_MOESM4_ESM.docx]

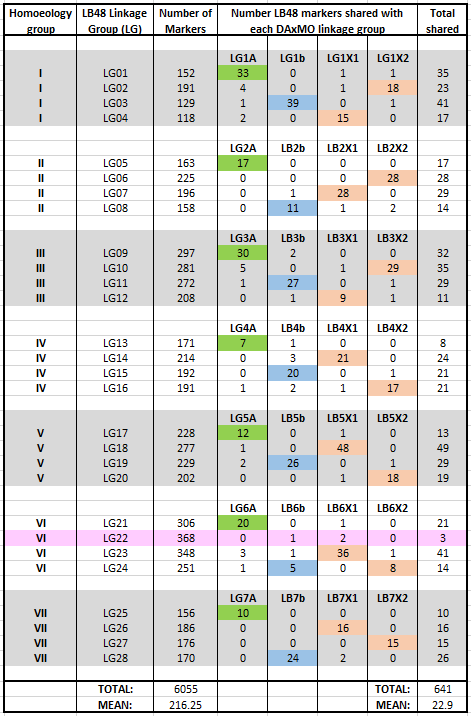
**Supplementary Table S3. Comparison between LB48 and DA×MO linkage maps.** Highlighted (green, blue, or orange) cells mark the intersection between an LB48 linkage group (rows) and the corresponding DA×MO linkage group. The shared marker counts for the seven DA×MO linkage groups identified by Sargent et al (2016)^19^ as comprising the A subgenome are highlighted in green. The shared marker counts for the seven DA×MO linkage groups provisionally identified ^19^ as comprising the “b” subgenome are highlighted in blue. As shown in Figure 4, part of LB48 LG24 corresponds to LG6b and part corresponds to LG6X2 of the DA×MO map. The row highlighted in pink is that of LB48 LG22, which does not have a corresponding LG in the DA×MO map.
